# A methodological review in sensory analyses of chicken meat

**DOI:** 10.1016/j.psj.2024.104083

**Published:** 2024-08-02

**Authors:** Matilde Tura, Mara Antonia Gagliano, Enrico Valli, Massimiliano Petracci, Tullia Gallina Toschi

**Affiliations:** ⁎ Department of Agricultural and Food Sciences, Alma Mater Studiorum - Università di Bologna, Bologna 40127, Italy; † Interdepartmental Centre for Industrial Agrofood Research, Alma Mater Studiorum - Università di Bologna, Cesena 47521, Italy; ‡ Department of Agricultural and Food Sciences, Alma Mater Studiorum - Università di Bologna, Cesena 47521, Italy

**Keywords:** sensory analysis, quality, descriptive sensory method, consumer test, poultry

## Abstract

The sensory characteristics of poultry products are crucial in defining their quality and widely influence consumer choices. Even though the scientific literature clearly indicates that for muscle foods the sensory profile is relevant in purchase decisions and overall acceptability, sensory evaluation has often been underestimated and considered complementary to instrumental and/or chemical assessments. Sensory analysis includes different types of validated tests (discriminative, descriptive, and affective), applied depending on the purpose of the research study, requiring special attention in the sample preparation phase, in particular for nonhomogeneous products such as poultry meat, requiring reproducible cutting, cooking and presentation to the tasters. The aim of this paper is to review, critically assess and discuss sensory methods, standardized procedures and sample preparation tailored for chicken meat, through the literature from 2000 to 2023, with a section dedicated to ethical aspects that must be carefully considered when designing a sensory protocol. The target readers are both the research and the business communities, as the information can be widely applied for quality control, to develop new food products, to understand or drive preferences or, for example, to assess potential sensory differences among chickens fed with different diets. To the best of the authors' knowledge, this review represents a useful first guide for those approaching the sensory analysis of chicken meat.

## INTRODUCTION

Consumers’ acceptance of raw and processed muscle foods is strictly related to their sensory attributes (Pateiro et al., 2022). Several authors reported that different factors can influence sensory assessments of meat; for example, the availability and familiarity of foods can affect the sensory evaluation (Sveinsdóttir et al., 2009). It has also been noted that easy access, product purchase frequency, and ethnicity influence the sensory preferences of meat (Prescott et al., 2001; Dyubele et al., 2010). Moreover, familiarity plays a key role, in fact, differences in perception of sensory attributes between consumers from different countries have been previously reported (Sañudo et al., 2007). Other factors can influence the sensory profile and characteristics of chicken meat, such as color, handling, exposure to chemicals, storage and cooking methods (Barbut, 2001; Fletcher, 2002). Thus, for poultry companies, it is essential to understand factors that affect the composition of chicken meat and drivers of preference. On the other hand, the knowledge of such factors is still limited in the current poultry production system (Worch et al., 2010).

Thus, poultry meat sensory quality and consumers’ preference and liking play a crucial role depending on several factors related to the bird genotype, sex, feeding, farming systems, age at slaughter and slaughtering procedures (Baéza et al., 2022). Several sensory methods can be applied, such as descriptive, discriminative or affective, depending on what the objective of the study is. In particular, descriptive methods (Descriptive Analysis, **DA**) are generally applied to characterize poultry products, as well as to identify and quantify different descriptors of their sensory profile (Lyon et al., 2007; Pateiro et al., 2022). On the other hand, to assess if two or more food products are different, it is advisable to select discriminative tests (**DT**) while to assess consumers’ preferences and liking the affective ones (**AT**) are generally used. In fact, although trained panels are generally preferred to describe the sensory profile and characteristics of meat, it has been also demonstrated that consumers can describe them in a reliable and repeatable way (Lyon et al., 2007; Worch et al., 2010). The present review aims to give an overview of the main sensory methods applied for the evaluation of poultry with special emphasis on chicken meat (from the year 2000 to now), according to different objectives, in order to give specific methodological indications and references to the readers.

## ETHICAL APPROVAL

Before starting any sensory protocol, considering the involvement of humans, it is mandatory to consider the *Declaration of Helsinki* and its requirements (The World Medical Association, 2008); thus, the experimental protocol has to be submitted for review by an ethical committee (**EC**). The EC could be described as an independent committee of people, e.g. professors, researchers, technicians, etc., who determine if trials involving humans are ethical. Its review is generally based on the reasons for conducting the test, the protocol, safety information, information given to the assessors, the recruitment plan, and other specific information related to the research plan (Kemp et al., 2011). Since the legal and ethical requirements for research are different from one country to another, the ethical committee in its review considers the laws and regulations of the country (or countries) in which the study will be performed as well as international norms and standards that are applicable to the specific research (Eccles et al., 2011). Human subject is identified as ‘*a living individual about whom an investigator obtains:* 1) *data through intervention or interaction with the individual, or* 2) *identifiable private information*’ (Eccles et al., 2011). The ethics review is required mainly because research involving humans could put people at physical, psychological, social, economic, legal, or dignitary risks or a combination of them, also considering that their exposure to risks is generally for the benefit of others. For this reason, it is needed an independent ethics review. This is because, although the integrity of researchers represents a protection for those participating as research subjects, the researchers themselves may not, however, be able to express the best judgment on the ethical acceptability of the study in question. Thus, any study that involves: 1) intervention on people; 2) interaction with them (e.g., interview or administration of a questionnaire); 3) collection of identifiable private data to contribute to knowledge must be submitted to an ethics committee. Therefore, for studies which involve interaction with human subjects or the collection of sensitive data or identifiable private information, ethical review is required. Indeed, the conduct of research involving human subjects requires independent judgment by a legally constituted ethics committee and this is not a judgment that can be safety made by researchers. What is expected is that ethics committee makes a disinterested decision, guided by the type of study, about the balance of benefits and harm to research subjects, the need for informed consent, and the need for other protections (Eccles et al., 2011). Informed consent must be collected from each subject participating in the study before starting the study itself; this is necessary because it represents the official document indicating that they are fully informed about the nature of the experiment, the samples they will ingest/use, and any associated risks, and on the fact that they can withdraw their participation at any time, as well as on the confidentiality of data collection. Several points need to be considered: 1) voluntary consent is essential; 2) subjects must have the legal capacity to give consent; 3) participants can exercise free power of choice; 4) subjects should have sufficient time to read and understand the information provided before providing their written consent and participation in the study (Kemp et al., 2011).

## SAMPLE PREPARATION

**Figure 1:**
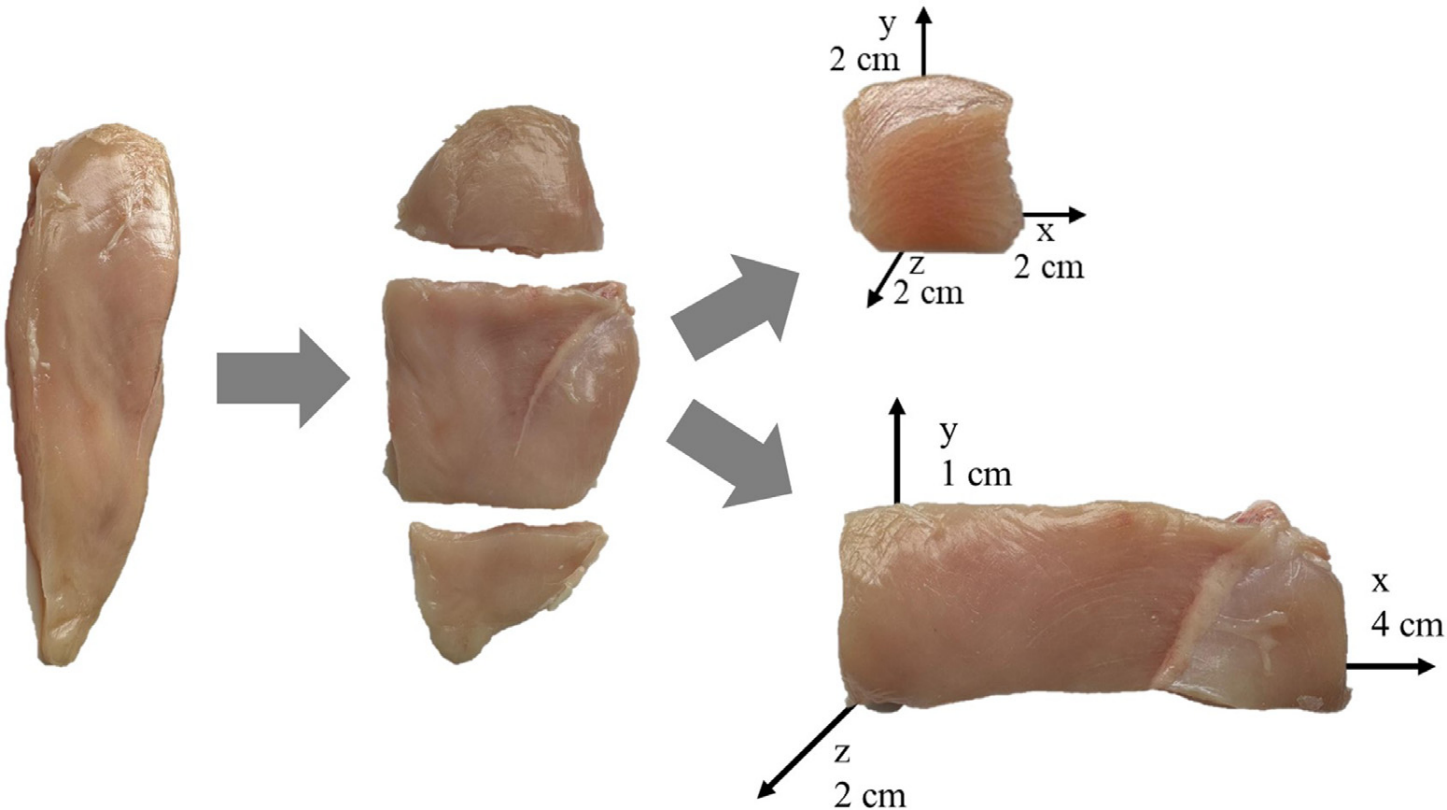
Image depicting possibilities of cutting a chicken breast sample for sensory evaluation, to achieve reproducible results.

From 2000 to 2023, many studies have been published in which samples of different parts of chicken are subjected to sensory analysis (Table 1). Several authors reported storing samples under refrigeration or freezing conditions and defrosting at 4°C to perform the sensory test (Dal et al., 2021; Shaviklo, 2023); however, it would be better if freezing was carried out rapidly at very low temperatures, <-20°C, in order to limit the damage resulting from the formation of ice crystals. Moreover, it is crucial to ensure that all samples freeze in the same amount of time. To achieve this, avoid stacking the samples in the freezer, as this would lead to varying freezing times, instead, arrange the samples in a single layer. If it is possible, meat samples should be freshly sensory assessed to avoid changes due to freezing (AMSA, 2016). In fact, as previously reported in literature (Zhang et al., 2020), the thawing process of chicken meat can affect several sensory characteristics, in particular related to the texture, such as juiciness and hardness. This influence on texture characteristics may be due to the fact that in frozen and thawed meat, the texture properties are affected by the combination of structural integrity loss caused by ice crystal formation and liquid loss during thawing (Zhang et al., 2020). Depending on the purpose of the research study, it is necessary to choose an appropriate protocol for sensory evaluation, including aspects ranging from product sampling to sample preparation and presentation. To evaluate raw meat, cooking procedures are generally envisaged, such as braising, grilling, electric grilling, roasting, broiling, outdoor grilling, and sous-vide cooking. For example, sous-vide cooking is applied to identify little differences in terms of flavor, avoiding the development of aroma resulting from the Maillard reaction (Mörlein, 2019). Also, the amount of sample presented to panelists for a test can differ substantially, for example, from presenting the whole muscle to small cubes of meat; anyway, the sample size should be enough to allow the evaluation by panelists and it has to be representative of the product (AMSA, 2016; Mörlein, 2019) (Figure 1). Moreover, it is essential to keep in mind sensory fatigue and satiety, avoiding obliging assessors to taste too many samples. For any type of sensory test, the order of presentation must be randomized and balanced to avoid first-position effects and carryover effects; otherwise, especially for consumer studies, a systematic bias of scores for the first sample can frequently occur (Mörlein, 2019).

**Table 1:** Summary of sample preparation conditions and serving procedures related to sensory evaluation of chicken meat from 2000 to 2023.

Product	Sample preparation and cooking	Serving procedure	Reference
Thighs	Boiling: • Covered with skin in saltwater (100° C, 15 min); • Heating 1h using a gas burner (internal temperature 85°C). Vacuum-packaged and boiling: • Vacuum-pack samples and store for 2 months at -20°C, thaw in a cold-storage chamber (1 d at 5°C), heat by keeping the sealed diffusion-tight plastic bags in hot water (80°C, 50 min; core temperature 80°C). Oven: • Cook directly (combi oven) from the frozen state to 77-78°C (12-15 min at 85°C). • 170°C in a convection oven (∼15 min, internal temperature 75°C). • Conventional oven (130°C, 20 min, internal temperature 80°C). • Dry-heat convection oven, 20 minutes at 240°C; Grill: • Electrically grill (40 × 20 × 10 mm), internal temperature 72°C.	Sample dimension: • 2 × 3 × 1.5 cm; • Two 1.9 cm squares. Serving temperature: • Warm in oven (60-70°C or 40±5°C), max holding period of 1 h after cooking. • ∼60°C.	Capita et al., 2000; Skrede et al., 2003; Jung et al., 2011; Zhuang and Savage, 2011; Smaoui et al., 2012; Bae et al., 2014; Francesch and Cartañà, 2015; Khan et al., 2016; Özünlü et al., 2018;
Breast	Oven: • Encased in aluminium foil in individual aluminium trays; 185°C 1.5 h (withdrawn skin after cooking); • Wrap samples (∼20 g) in aluminum tin foil, in roasting trays, 180°C in a fan-assisted oven for 30–35 min. • Preheat an electric oven to 190°C, samples in uncovered pans, to an internal temperature of 74°C; • Roasted in a convector oven, 180°C, internal temperature of 80°C; • Put in aluminium foil-covered pans in an air convection oven (176°C to internal temperature 76°C); • Dry cooked at 170°C in convection oven (15 min, to internal temperature 73°C); • Convection oven, fillets in pans covered with aluminium foil to internal temperature 76°C; • Covered samples with foil, preheated convection oven at 177°C (rotate trays for uniform cooking, internal temperature of 76.7°C); • Samples in polyester film, heat in a conventional oven (200°C for 1 h, internal temperature of 80°C); • Combi oven to the endpoint temperature of 78°C; • 10-13 min to an internal temperature of 78-80°C. • Fillets (with skin) in preheated fan ovens (180°C to core temperature 75°C); • Oven at 177°C to an internal temperature of 77°C; • Convection oven, 20 min industrial cooking program (dry heating for 5 min, steam cooking for 10 min and dry heating for 5 min), internal temperature 74°C; • Air-convection oven, internal temperature 72°C; • 176.67°C (350°F) convection oven, internal temperature 74°C; • 177°C, convection oven, internal temperature of 73°C; • Convection oven, 180°C, turning every 3 min, international temperature 71°C; • 180°C for 15 min in a preheated oven; • Samples, covered with aluminium foil, in oven, 105°C for ∼60 min, turning samples every 10 min. • 205°C, internal temperature of 77°C; • Roast the pectoral whole muscles, convective-steam oven at 180±2°C, internal temperature 75 ± 1°C in the geometric center of the muscle; • In aluminium foil at 180°C for 20 min; • Electric oven at 200°C with regular turning of samples; • Cook fillets in vacuum sealed bag in a combi at 83.88°C, endpoint temperature of 76°C; • 180°C for 15 min in preheated oven; • Combi oven, 100°C with 100% heat, 20 min (core temperature 72°C); • 80°C for 1h; • Forced draught oven, 180°C to core temperature 75°C; • 2 cm^3^ samples in a dry oven at 120°C, 30 min; • Grill in a kitchen oven for 40 min at 190±5°C with a core temperature of 75°C; • Cook samples in a combi oven on a metal pan in a single layer at 85°C and tender steam to an endpoint temperature of 80°C. Sous-vide: • Vacuum pack individually, sous vide method, internal temperature of 78°C, in a circulating water bath; • Pack samples in cook-in plastic bags (sous-vide method), cook in hot water at 85°C- 95°C, core temperature 72°C; • Vacuum-packaged bags by immersing in water at 85°C to internal temperature of 80°C; • Vacuum-packed, 10 mm thick slices, for 50 minutes at 85°C in a water bath with no spices or additives; • Individually vacuum-packed in polypropylene bags, in a water bath set at 80°C, core temperature of 78°C; Microwave: • Heat samples in the microwave at high power (700 W) for 4 min including the time of defrosting; • Roast 2 cm cube samples in a microwave oven at high power (800 W) for 30 s; Cooker: • Seal samples in a plastic bag and cooked in a cooker without pressure for 20 min, internal temperature 88°C; Grill: • Wrap the fillets in aluminium foil and cook on an electric grill to an internal temperature of 82°C. • Grill (1 cm thick) on both sides for ∼45 s, internal temperature 71 to 75°C; • Cook from freezing to an internal temperature of 74°C using a grill; • Grill on a kitchen pan at around 70°C, for 5 min; • Grill breast slice (80 g, 1 cm thick) at 300°C for ∼7 min, internal temperature 72°C; • Wrap in aluminium foil and bake on a preheated electric grill until an internal temperature of 82–85°C; • Grill on both sides 1 cm thick portions for ∼45 s, internal temperature of 71-75°C; • Cook to an internal temperature of 73°C using either a flattop grill set at 177°C; • Grill (0.5 kg, thickness ∼1.5 cm) for 40 min, turning samples every 3 min without oil/seasoning; • Grill for 1 min for each side in a grill pan; Roasting: • Electric roaster, cooking at 160°C for 1 h 15 min; • Roast on a wire mesh placed on an open braai stand for 30 min; • Cooked on a commercial rotisserie, internal temperature of 80°C; • Boiling/cooking in water: • Boil in water using an ordinary kitchen stove (salt added) for 30 min; • Packed in individual plastic bag and sealed and immersed in a water bath at 80°C for 5 min; • Boil in a water bath for 15-25 minutes at 80°C; • Heat in water baths set at 53°C or 58°C for 45 min; • Boil on a hotplate in stainless steel pot in 1:1 (w/v) ultrapure water for 20 min at 120°C, to an internal temperature of 85–95°C; • Heat in water with sodium chloride (1:1.5:0.01, w/v/w) at 100°C for 1 h using a gas burner and cook to an internal temperature of 85°C; • Cook individually in a covered container in 400 mL of 0.6% saline, until inside temperature of 70°C; • Cook for immersion in a hot water (80°C) bath for 50 min until a core temperature of 76°C; • Boil for 20 min in water (water: diced breasts, 2:1, m/m); • Cook (400 (±15) g of breast muscles without skin and internal fat, vacuum-packed) immersed in a water bath at 75°C in a circulator under cover for 40–75 min until internal temperature of 72°C. • Others: • 10 g pieces of sample cooked to an internal temperature of 80°C. • Cook in an ordinary kitchen stove at a temperature of 70°C; • Cook for 10–15 min in each evaluation step with a home steamer pot;	Sample dimension: • Cut parallel to the muscle fibers into 1 × 1 x 2 cm strips; • 25 (±5) g; • Section into cubes; • After cooking cut into half-inch cubes; • Trimmed external connective tissue and cut into ∼20 mm in length; • Cut into small cubes 3 × 1 × 4 cm; • Trim the central portion of each breast to ∼5 × 6 cm; • 1 × 1 × 1 cm cubes; • ∼2.5 cm in length pieces of cooked breast; • Cut in a 1.9-cm-wide strip parallel to fibers; then cut into 2 cubes of 1.9 cm trimmed as needed the bottom of a strip to ensure uniform sample cubes; • Cut into 1 cm thick slices (1 slice per assessor); • Cut into 2.5 × 2.5 × 2.5 cm cubes; • 1.25-cm^2^ warm cubes; • 80 g for trained panellists and 20 g for consumers; • 5 cm cubic pieces; • 2 × 2 × 2 cm (width × length × height); • Cut parallel to the muscle fibres into 1.5 cm cubes; • Rest samples for 3 min before slicing for sensory testing. Remove the tendon end by making a perpendicular cut. Remove a 1.9 × 3.8 cm strip and cut the 3.8 com length into two 1.9 com pieces for sensory analysis. • 2 × 3 × 1.5 cm; • 2.5 cm length 9 1.5 cm width 9 1.5 cm height; Serving temperature: • Close samples in individual plastic containers and heat in a 700 W microwave oven for 20 s and present warm; • Place samples in a closed plastic bag and suspended in 60°C water for 5 min before sensory analysis; • Samples cooled to 43°C at room temperature; • Conserve samples in a hot bath at 60°C; • Kept warm in oven at 35-40°C until served; • Place samples in a souffle cup; • Samples cooled to 60°C; • Immediately distributed after cooking; • Served at a temperature of 100°C; • Cooled samples to 50°C; • Wrap in aluminium foil, keep at 60°C; • Serving temperature of about 55°C; • Served under an aluminium cover on a pre-heated plate; • Served the fillets on a 60°C hot plate 2 min after they were cooked; • Cool at room temperature for 15 min, kept warm (60-70°C) in 7.6 L chafer dishes; • Presented warm (75°C) to the assessors; • Served samples warm (minimum 60°C; within 20 min after cooking); • Serving temperature of 50°C; • Keep samples at 50°C in the water bath until served; • Cool cooked samples for 3-5 min, cut samples in slices and present two 1.9 cm squares of meat from each sample. Serve samples in capped 4 ounce Styrofoam; • Samples at 25°C; • Cooled samples at room temperature for 15 min and kept warm (60-70°C); • Samples at 40-45°C; • Cool samples at room temperature for 25 min (i.e. ∼50°C); • Serve samples at 65°C;	Gonzalez-Esquerra and Leeson, 2000; Schilling et al., 2003; Kennedy et al., 2004a; McNeill et al., 2004; Sen et al., 2005; Dyubele Rababah et al., 2005; de Toledo et al., 2005; Balamatsia et al., 2006a; Balamatsia et al., 2006b; Chouliara et al., 2007; Balamatsia et al., 2007; Contreras-Castillo et al., 2007; Jaturasitha et al., 2008; Lee et al., 2008; Chouliara et al., 2008; Yusop et al., 2009a; Yusop et al., 2009b; Saha et al., 2009a; Saha et al., 2009b; Zhuang et al., 2009 Yusop et al., 2010; Sow and Grongnet, 2010; Dyubele et al., 2010; Kruk et al., 2011; Lee et al., 2011; Vaithiyanathan et al., 2011; Erwan et al., 2011; Cross et al., 2011; Chulayo et al., 2011; Jeong et al., 2011; Broadway et al., 2011; Sampaio et al., 2012; Zhuang and Savage, 2012; Christensen et al., 2012; Horsted et al., 2012; Schilling et al., 2012; Yusop et al., 2012a; Yusop et al., 2012b; Kamboh and Zhu, 2013; Sanchez-Pena and Alvarado, 2013; Khiari et al., 2013; Napolitano et al., 2013; Casco et al., 2013; Gopinger et al., 2014; Jung et al., 2014; Kruk et al., 2014; Schilling et al., 2015; Brambila et al., 2016; Motsepe et al., 2016; Khan et al., 2016; Galarz et al., 2016; Tasoniero et al., 2016; Ghollasi-Mood et al., 2017; Haščík et al., 2017; Trembecká et al., 2017; Brambila et al., 2017; Lytou et al., 2017; Lytou et al., 2018; Michalczuk et al., 2018; Chumngoen et al., 2018; McLeod et al., 2018; Gunya et al., 2018; Aguirre et al., 2018; Brambila et al., 2018; Marcinčák et al., 2018; Augustyńska-Prejsnar et al., 2018; Orlowski et al., 2018; Orczewska-Dudek and Pietras, 2019; Kerdpiboon et al., 2019; Toomer et al., 2019; Sengun et al., 2019; Hussein et al., 2019; Damaziak et al., 2019; Pieterse et al., 2019; Park et al., 2020; Zhang et al., 2020; Hudák et al., 2021; Fedorov et al., 2021; Pettersen et al., 2021; Pietras et al., 2021; Abdel-Naeem et al., 2021; Adeyemi, 2021; Liu et al., 2021; Shaviklo et al., 2021; Escobedo del Bosque et al., 2022; Piruz and Khani, 2022; Hailemariam et al., 2022; Abdel-Naeem et al., 2022; Panahi and Mohsenzadeh, 2022; Konkol et al., 2023; Barazi et al., 2023; Nemauluma et al., 2023; Andaleeb et al., 2023; Jeong et al., 2023;
Leg	Oven: • Roasted in a conventional oven, 190°C, internal temperature 82-85°C. • Convection oven (200°C, 60% humidity, 1 h); • Pre-heated oven (180°C), core temperature 80°C;	Serving temperature: • Keep samples in a conventional oven at 75°C to maintain the temperature until serving (residence time of sample inside the oven never exceeded 15 min). • Covered with aluminium foil and held in an oven at 77°C until served.	Bou et al., 2001; Mahrour et al., 2003; Khan et al., 2016; Šťastník et al., 2018; Yasar et al., 2018;
Entire carcass	Oven: • Wrapped in aluminium tin foil, placed in Pyrex roasting dishes, cooked for 2 h at 200°C using a fan-assisted oven, rotate every 40 min (min. internal temperature 80°C).	Sample dimension: • Remove the left and right breasts from the chicken carcasses and portion; Serving temperature: • Cover samples in aluminium foil and hold on a bain marie at a temperature of ∼70°C before serving.	Kennedy et al., 2005;

## SENSORY METHODS TO EVALUATE CHICKEN MEAT

### DESCRIPTIVE SENSORY ASSESSMENT OF CHICKEN MEAT

**Figure 2:**
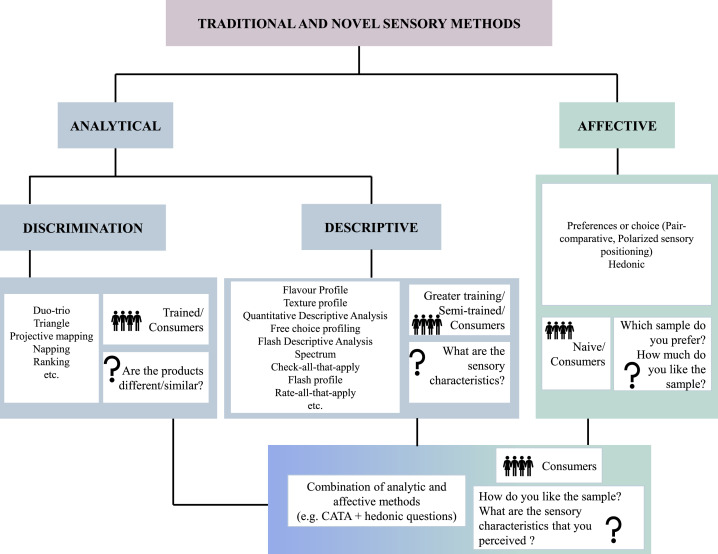
Overview of the sensory methods that can be applied for the evaluation of chicken meat, with the indication of the level of training of the assessors and the question to which each test category answers, adapted from Ruiz-Capillas et al. (2021).

Descriptive tests involve a comprehensive sensory analysis of products and require a trained sensory panel for accurate results, which can be quantified (Figure 2). In these analyses, it is crucial to identify descriptors that offer maximum information about the sensory properties of the product. Panelists evaluate their perceptions using quantitative values proportional to the intensity of specific attributes. To achieve significant results, panelists must undergo rigorous training. While traditionally conducted by fully trained panels, certain novel sensory techniques allow for the involvement of semi-trained panelists. These methods broaden the applicability of descriptive tests, potentially making them more accessible and efficient in certain contexts. Various descriptive methods, including the flavor profile and the texture profile ones, rely on trained judges. For instance, the texture profile method is employed to discern specific intensities in a product by utilizing control products. A notable advancement in these methods, applicable not only to taste and texture but to various attributes, is the Quantitative Descriptive Analysis (**QDA**). Other descriptive procedures, such as free-choice profiling, flash descriptive, and the spectrum method, also contribute to a more nuanced understanding of product characteristics. Structured and equidistant scales are commonly utilized in descriptive analysis. Within these scales, panelists assess their perception of a specific attribute and assign it a determined intensity. This systematic framework is essential for capturing and quantifying sensory attributes, contributing to enhanced precision and comparability in descriptive analyses. The strength of the attribute is typically represented on a horizontal scale, often marked vertically, facilitating the assessment of its numerical assignment. These scales can be designed for a single attribute or multiple attributes and descriptors, creating a descriptive profile of the products, as seen in QDA. The arrangement of descriptors within these scales follows a logical order of perception, encompassing sight, smell, and sensation in the mouth. The careful selection of descriptors is crucial in these analyses. They must accurately convey the sensory impulse, be specific and clear in describing the sensation, and possess relevance and discrimination power pertinent to the products under analysis. This meticulous choice of descriptors ensures the effectiveness of the descriptive analysis process. In general, these scales offer advantages such as the use of fewer tasting samples and a smaller number of trained tasters, although the potential for fatigue errors does exist. However, when employing semi-trained tasters, a challenge arises due to the excess of parameters under evaluation, potentially leading to difficulties in discerning between very similar parameters. This complexity may result in a loss of interest among semi-trained tasters, negatively impacting the final results. Despite these challenges, descriptive analyses are generally considered among the most suitable sensory tests. They not only require less extensive training but also provide a substantial amount of information. The results are easily interpretable, making descriptive analyses valuable tools (Ruiz-Capillas et al., 2021).

### Conventional Descriptive Tests

Sensory attributes, encompassing features such as appearance, odor, flavor, taste, and texture, which are perceptible by the human senses, play a vital role in assessing the quality of muscle foods. These characteristics often serve as benchmarks during the selection of food items. Conventional descriptive sensory analysis stands as an analytical method in sensory evaluation, involving the discernment and detailed description of sensory components in products by a trained panel. The panelists undergo screening and training to assess specific characteristics based on discrimination and description analyses. The trained panelists are expected to proficiently identify and quantify specific attributes, offering insights into both instrumental and sensory measurements of foods (Chumngoen and Tan, 2015). The BS BS EN ISO 13299 (2016) reported several general instructions to perform sensory profile methods. Firstly, the tests should be carried out in the laboratory which should be equipped with the necessary tools for preparing samples (BS EN ISO 8589:2010). The person designated to perform the sensory profile tests is called the “panel leader” and he/she is in charge of training assessors, maintaining the training of the panel, and executing the test. For sensory descriptive tests, the number of assessors as well as their level of training should be adapted to the specific method. Enhancements in repeatability and reproducibility are achieved through the selectivity of assessors and the duration of their training. The understanding of results and the discernment of notable distinctions among products are likewise contingent on the number of assessors and the extent of their training. The recruitment of candidates is typically conducted through presentations, circulars, or personal contacts (BS EN ISO 13299, 2016). Interviews and screenings have to be conducted for two to three times the necessary number of assessors. During this process, key considerations include: good health compatible with product testing, demonstrated interest and motivation, commitment to the agreed-upon duration and availability for panel sessions, promptness in fulfilling responsibilities, capacity to concentrate effectively, memorization skills, honest communication and reporting of sensations, discrimination ability regarding studied characteristics, and capability to collaborate and work well in a group (BS EN ISO 13299, 2016). To achieve balanced sensory acuity, panels consisting of 10 or more assessors should be established. The study's products and their preparation conditions must be clearly defined. Special precautions must be taken to prevent assessors from deducing conclusions about the sample nature based on presentation. For instance, the use of colored testing glasses or lights can be implemented to mask differences in appearance, if necessary. Standardization is essential in the preparation and distribution of samples, ensuring a uniform temperature. Each sample will be assigned a three-digit random code, and the order of presentation will be meticulously defined through an appropriate design (BS EN ISO 13299, 2016). To enhance the reliability and validity of results, each sample or sample group should ideally be presented two or three times, or more if possible, and on different days. The decision on the number of replications should consider the required precision, observed result dispersion, and any discernible trend towards improved discrimination as assessors become more acquainted with the samples. Replication helps estimate experimental error. Assessing a product from the same batch multiple times illustrates the dispersion of scores given by one assessor while assessing a product from different batches reflects variations within the product. The protocol should specify which sample(s) are duplicated and under what conditions they are prepared and assessed. The identity of the samples should remain undisclosed until assessors complete all assessments (BS EN ISO 13299, 2016). There are several conventional descriptive methods (i.e., consensus profile, quantitative descriptive analysis, flavor profile, texture profile). One of the most used tests is Quantitative Descriptive Analysis (**QDA**). Quantitative Descriptive Analysis emerged in the 1970s as a response to perceived issues associated with Flavor Profile Method (**FPM**), as discussed by Stone and Sidel (1993) and Stone et al. (1974). Notable distinctions exist between FPM and QDA. In QDA methodology, participants were recruited from sources external to the project. These individuals underwent screening involving dietary questionnaires and the products under test, with the premise that frequent consumers of the product were more sensitive to product differences, rendering them more discriminating (Murray et al., 2001). In QDA, the language source is deliberately non-technical and reflects everyday language. This approach is adopted to prevent any bias in response behavior that might arise from providing specific language, which could imply correct or non-correct answers. Reference standards are introduced in QDA only when there is a recognized issue with a particular term. Subjects usually require references in only 10% of cases, as discussed by Stone and Sidel (1993). In fact, after the recruitment of the assessors, the first phase of QDA as well of the most conventional descriptive sensory profile methods, is the training of the assessors. Firstly, the training phase of descriptive sensory analysis starts with the establishment of a shared vocabulary that thoroughly and precisely describes the attributes of the product (Murray et al., 2001). The overall objective is to identify and choose a set of attributes that are non-overlapping, singular, objective, unambiguous, and referenced. These attributes should enable to the greatest extent possible a comprehensive descriptive analysis of the samples under investigation. This crucial step can be performed either individually or collaboratively, depending on the adopted sensory profiling method (i.e., list of attributes needs to be common for QDA). If a unified list is required, the panel leader can utilize one of the three approaches outlined in Table 2, or a combination of them.

**Table 2:** Different approaches for the definition of the list of sensory attributes for a specific food product (BS EN ISO 13299:2016).

Approach	Method	Advantages	Disadvantages
Use existing terminology and reference standards.	Seek guidance from literature and experts for a suitable selection. Obtain the specified standards and use them to instruct assessors on the quality of each descriptor, and if necessary, provide an intensity scale for that descriptor.	Profiles can be interpreted differently by different panels and compared to other research findings.	While existing terminology or reference standards may be available, they might include choices that are imprecise or unsuitable for a specific set of samples. This approach may overlook attributes that could have been identified through the development of new terminology.
Conduct special sessions with the panel to collaboratively develop the terminology.	Employ a panel of chosen assessors and facilitate the development of terminology through round table discussions led by an experienced panel leader. Reference standards, provided by the panel leader, the test requester, or an assessor during the session, are utilized.	The process of terminology development is not so time-consuming.	Profiles obtained are distinctive to a specific panel and set of samples, making interpretation challenging for other groups without reference standards.
Conduct special sessions with the panel to collaboratively develop the terminology.	Identification and selection of discriminating terms by employing a set of prepared training samples. Subsequently, reduce the number of terms through stepwise elimination using statistical techniques.	A completely objective process of selection and elimination is employed, minimizing terms influenced by traditional misconceptions or preconceived notions. The chosen terms aim to provide optimal coverage of the qualities perceived by assessors in the samples.	Profiles acquired are specific to a particular panel and set of samples, making interpretation challenging for other groups without reference standards. The process is somewhat time-consuming and demands a certain level of expertise, particularly in data analysis.

Typically, a new established sensory panel creates its own sensory language, but guidance from an experienced panel leader or other members of the organization can facilitate the learning process. Alternatively, an existing language may be adopted, although challenges in understanding and interpreting terms may arise if the language has been developed by another laboratory, or in a different country or region. To address this issue, providing comprehensive definitions and standards can ensure clarity in demonstrating the sensory attributes (Murray et al., 2001). Moreover, the order of perception in which the attributes are evaluated must be set up (e.g., appearance first, aftertaste last) (BS EN ISO 13299:2016). Once the common vocabulary has been defined, the quantitative training of the assessors has to be performed to indicate the intensity of each attribute present in the sample. Thus, a response scale must be selected. Generally, this scale may be numerical or semantic, continuous, or discontinuous, unipolar or bipolar (BS EN ISO 13299:2016). In order to perform both qualitative and quantitative sample evaluation, a pre-printed scoresheet containing attributes and selected scale shall be prepared and used; it is recommended to leave a blank space on scoresheets and prompt assessors to provide comments or suggestions for additional attributes (BS EN ISO 13299:2016). During quantitative training, as well as during the evaluation of samples, assessors have to work alone in individual sensory booths and samples have to be presented monadically (in succession, one-by-one) in randomized and balanced order (BS EN ISO 13299:2016). Moreover, it is necessary to adjust the number of samples per assessor and per session, based on factors such as the session's duration, the nature of the products, the number of attributes, and the anticipated differences. It is also strongly recommended to present a limited number of samples when small differences are expected, especially for poultry products with strong or persistent flavors (BS EN ISO 13299:2016). Statistical analysis is required when expert and trained panels are involved. In particular, the interpretation of results involves 3 steps: 1) the first step to assess the performance of assessors and checks for any experimental errors in the data, it is generally conducted using ANOVA; 2) the second step is commonly referred to as univariate analysis and it focuses on each evaluated descriptor, aiming to identify the descriptors that effectively discriminate among the study's products; the third step considers all descriptors deemed useful in the initial stage, often known as multivariate analysis; it can be used spider graphs to represent the sensory profile of the samples. The latter can be executed following the segmentation of descriptors, such as visual, flavor, taste, and texture descriptors (Murray et al., 2001; BS EN ISO 13299:2016). One limitation of QDA is the difficulty in comparing results between panels, across different laboratories, and from one period to another using this technique; moreover, it is time-consuming (Murray et al., 2001).

Several authors adopted descriptive tests by using trained panels for assessing the sensory profile of chicken meat (Ruiz et al., 2001; Liu et al., 2004; de Toledo et al., 2005; Zhuang and Savage, 2010; Chumngoen and Tan, 2015; Franke et al., 2017; Aguirre et al., 2018; Brambila et al., 2018; Siekmann et al., 2018; Escobedo Del Bosque et al., 2020; Katiyo et al., 2020; Pellattiero et al., 2020). Some studies aimed to evaluate the main sensory characteristics (e.g., appearance, flavor, and texture) of chicken meat as affected by genotype (Chumngoen and Tan, 2015) and wooden breast condition (Aguirre et al., 2018), while other authors investigated possible changes related to dietary supplementation with antioxidants (Ruiz et al., 2001).

### Rapid Descriptive Tests

As previously mentioned, a significant drawback of QDA lies in the substantial time investment required for training, coupled with challenges in validating the panel, especially when dealing with samples that lack standardization. In such cases, samples can exhibit heterogeneity and pose challenges to repeatability over time. To address these constraints, rapid descriptive methods have been recently developed. These methods encompass evaluations of individual attributes, such as Free Choice Profile (**FCP**), Intensity Scales (**IS**), Check-all-that-apply questions (**CATA**), Flash Profiling (**FP**), and Paired Comparisons. Additionally, methods based on the assessment of global differences, such as Sorting, Projective Mapping (**PM**), or Napping, as well as those relying on a free, overall evaluation of individual products through open-ended questions, have gained prominence. These methods can employ either a semi-trained panel or untrained assessors (consumers), serving as important tools for food development and quality control. They find extensive applications in Food Science and Technology (Aguiar et al., 2019). Among them, the flash profile is commonly used. Flash Profile (**FP**) is a technique originally proposed by Dairou and Sieffermann (2002) and it combines elements of Free Choice Profiling and Ranking Descriptive Profiling. When using this approach, consumers are firstly tasked with individually identifying attributes and then ranking samples based on their intensity, all without prior training. Due to varying sets of attributes per assessor, the data matrix is incomplete. To address this, Generalized Procrustes Analysis (**GPA**) is applied, resulting in a descriptive map reminiscent of Free Choice Profiling (**FCP**). In FP, the number of assessors typically ranges from 8 to 30. Aligned with other rapid methods that involve untrained assessors and often necessitate a larger panel size, recent studies have expanded the FP panel to include 18 and 30 consumers (Aguiar et al., 2019). FP can be performed in 5 sessions, in which in the first the individual lexicon is developed by each assessor, in the second one the assessors choose their definitive list of attributes and sessions from 3 to 5 are used to evaluate the products in triplicate. All samples are presented simultaneously to the assessors, who are then instructed to rank products for each attribute using an ordinal scale. The evaluation sessions lasted approximately 1 h (Dairou and Sieffermann, 2002).

Another commonly applied method is Check-All-That-Apply (**CATA**). This method relies on a pre-developed list of descriptors. Respondents are provided with an object to evaluate (e.g., a food or beverage product) and a list of terms to characterize it. Their task is to select all the terms they deem appropriate, and the relevance of each response option is determined by calculating its frequency of use (Ares and Jaeger, 2013; Aguiar et al., 2019). One of the primary advantages of CATA questions is that consumers perceive the task as easy and not tedious to complete. However, extending the list of terms in the CATA question can diminish the perceived ease of the task and increase its tediousness, potentially compromising attention. Consequently, lengthy lists may encourage satisficing response strategies, where consumers opt for terms that quickly catch their attention without deeply considering the sensory characteristics of the samples (Jaeger et al., 2015). It should be taken into account that CATA is generally applied with untrained subjects (consumers) thus it needs a high number of respondents (at least 60–100 consumers). Moreover, to avoid bias, the literature suggests randomizing the order of CATA options presented to respondents. This randomization should occur not only between respondents but also within respondents, ensuring different question orders for each respondent and different term presentation orders for each sample evaluation. Randomization aims to mitigate memory limitations, cognitive process effects, and the influence of attention on memory. Consistent exposure to responses in the same order may automatically shift consumers' attention to options used previously, both voluntarily and involuntarily (Ares and Jaeger, 2013). Xu et al. (2020) applied CATA method to assess the emotional responses to flavor of chicken meat samples. In particular, the authors recruited 61 consumers who had to select the emotional attributes from a 10-descriptor list (comfortable, relaxed, blissful, pleasant, surprised, satisfied, refreshing, annoyed, abhorrent, and lonely). Currently, to the best of our knowledge no studies using rapid descriptive tests are available in literature on poultry meat.

## DISCRIMINATION METHODS

Discrimination tests are analytical methods which are generally considered easy-to-apply sensory techniques to investigate whether participants can detect any sensory differences between two samples, while sensory profiles and descriptors are not evaluated (Figure 2). For this category of sensory tests, it is essential to eliminate the component due to chance in the analysis; for this reason, the number of participants must usually be high enough to be able to appreciate significant differences between the products (Ruiz-Capillas et al., 2021). On the other hand, discrimination tests do not require trained panelists or a high level of expertise (Rousseau, 2015). In fact, they are rapid tests that are easy to handle (simultaneous presentation), analyze and interpret, performed with experienced tasters or by consumers (but not by a combination of the two). This is because, generally, trained tasters are more sensitive than consumers due to their greater experience, resulting in a lower quantity of noise associated with the test, reducing the variance of the perceptual distributions (Rousseau, 2015). Usually, the most applied discrimination techniques are the paired-comparison method, duo-trio, and triangle test (Ruiz-Capillas et al., 2021). Also, in this case, randomization is needed to prevent an influence on the results due to the presentation order (Rice and Meullenet, 2012). They can be also often used as preliminary sensory evaluations, before descriptive or affective tests.

### Triangle Test

The triangle test is part of rapid discrimination tests and it is mainly used to determine whether there is or not a perceptible sensory difference between two products. It is a “forced choice” method, and panelists are required to respond even if they guess, it is not allowed to report “no difference” (BS EN ISO 4120, 2007; Sinkinson, 2017). Three samples, two of which are the same, are presented simultaneously to each participant/panelist who will have to identify the one that is different from the other two. This test can be applied to assess overall product differences, to determine the sensory effect of a change in formulation, packaging, processing, handling, or storage conditions or for screening/validating panelists during recruitment (Sinkinson, 2017). The method can be applied only if a little difference exists between the products; if the difference is large or easily noticeable, a discrimination test is not the appropriate methodology. It is necessary to highlight that the only differences between the products should be due to the modification being studied, if there are other variations, such as different production methods, different raw materials, inhomogeneities within the products, etc., it will not be possible with this methodology to identify whether the perceived differences between the products are due to the design change under study or to these other modifications. The main aim is to maximize the chances of finding a significant difference between two products for the factors of interest (BS EN ISO 4120, 2007). Moreover, in addition to having to be homogeneous, the products must be prepared and presented together identically; the same quantity must be served, and the samples must have the same size and the same temperature. This is to avoid the stimulus error whereby panelists would be influenced by other characteristics unrelated to the test, that is, panelists will be influenced by a difference in portion size, a difference in color or texture, or a temperature difference. No visual differences should be evident; if these exist, they must be masked using, for example, colored lights (e.g., red, or green). However, if the change being tested is visual differences, no further masking actions are necessary (Sinkinson, 2017). The panelists are informed that two are the same and one is different. They can evaluate the samples in a specific order (e.g., from left to right) to identify the 'different' sample and describe the differences among the samples on the accompanying sheet (AMSA, 2016) and that it is better to perform the evaluation quickly to compare samples effectively (BS EN ISO 4120, 2007). The presentation order of the sample must be randomized and balanced within the panel so that the 6 possible combinations of the two samples have to be included. If we consider product “x” as A and product “y” as B, the presentation order to compare them is AAB, ABB, ABA, BAB, BAA, BBA, thus, they are presented the same number of times. Thus, there are 6 possible combinations of sample presentation randomization, thus, to prevent psychological errors (e.g., central tendency) (Rice and Meullenet, 2012; Sinkinson, 2017). Moreover, it could be useful to provide appropriate devices for cleaning the oral cavity and 3-digit codes must be used to identify the samples (blind) (AMSA, 2016; Sinkinson, 2017). Participants can guess correctly only a third of the time (*p* = 0.333) and the statistical test to be applied is the one-tailed binomial test (Sinkinson, 2017). Processing of results is based on the minimum number of correct responses required for significance at a predetermined significance level, given the total number of responses received; the minimum number of correct answers can be found in the statistical tables (BS EN ISO 4120, 2007; ASTM E1885-04, 2007; Stone and Sidel, 2004). It is permissible to ask participants to further describe the nature of the sensory difference they perceived. If this descriptive information is also collected, only the indications of the tasters who answered correctly should be considered. This data, however, should not be analyzed but can only be used as qualitative information to identify trends to be investigated using descriptive tests (Sinkinson, 2017); this is because the triangle test does not provide indications regarding the direction of the difference, such as the identification of a specific sensory attribute (BS EN ISO 4120, 2007) or the magnitude/intensity of the difference. Furthermore, the test engineer should not be tempted to conclude about the magnitude of the difference from the significance level or probability (*p*-value) from the analysis (Heymann and Lawless, 2013). The analysis of the triangle test data is based on probability and the conclusion that can be drawn depends on the risk that the person in charge of the test is willing to take. The risks are:

• Alpha risk (or false positive): risk of concluding that a perceptible difference exists between the two products when in truth they are the same. It is essential to minimize this risk when the objective is to determine “a difference” between 2 products, in this case, it is generally applied α ≤ 0.05 (5%);
• Beta risk (false negative): risk of concluding that no perceptible difference exists between the 2 products when in truth they are different. It is necessary to be minimized when the objective is to determine the similarity of 2 products; in this case, the usual level of acceptable β-risk is < 0.05 (5%).

By setting α and β risks, the minimum needed to meet the confidence levels set for the test, that is, number of independent results (**N**), can be found using specific tables; if no replies are expected, this corresponds to the minimum number of panelists. In general, a larger number of assessors gives a greater degree of confidence but also determines higher resource costs (BS EN ISO 4120, 2007 Tables Annex A). When planning to carry out a triangle test for similarity, it is essential to also consider Pd, that is, the maximum percentage of “distinguishers” that the person in charge of conducting the test can tolerate being able to detect a difference among products (BS EN ISO 4120, 2007).

Du et al. (2002) applied the triangle test to investigate possible differences in terms of odor of the irradiated and nonirradiated broiler breast fillets, highlighting differences for the fillets stored under vacuum conditions before cooking while for the fillets aerobically stored before cooking the difference in odor decreased with the increase of the storage time (Du et al., 2002). McNeill et al. (2004) conducted a triangle test (N = 20) on chicken breast fillets to determine whether consumers could detect any difference in flavor between the samples, detecting difference between the control and the meat from the broilers fed on 200 g/kg rapeseed. Also, Nieto et al. (2020) performed a triangle test to investigate differences among chicken breasts immersed in hop extract vs. water, highlighting no statistical differences (N = 54 subjects; α = 0.1; β = 0.05; P_d_ = 30%).

## AFFECTIVE TESTS

Affective tests also referred to acceptability or hedonic, are used to evaluate the degree of satisfaction of a product based on its sensory appeal (Figure 2). This test is performed by untrained participants, typically consisting of more than 100 subjects, who are selected to use the product (Fiorentini et al., 2020). Affective tests gauge product preference, including preference analysis and consumers' willingness to pay, as well as the degree of acceptance through hedonic evaluation. Typically, panelists are considered as naïve consumers without specialized training in preference description. Their assessments rely on taste, centering on the purchase decision and overall acceptance. Preference or choice tests enable the determination of whether a product is preferred or not, relying on the predominant response from a panel. Including the "no preference" option is advisable, as it enhances the information available for interpreting results. These preference techniques, commonly utilized in market research for new products, offer valuable insights into diverse population segments. Despite their usefulness, a notable limitation lies in the fact that this methodology does not provide information about the degree of liking or disliking from respondents. Panelists are limited to expressing whether they like or dislike a product without indicating the intensity of their preference. To gather more comprehensive insights, hedonic tests prove instrumental. Employing the hedonic method involves evaluating the product's likability using hedonic scales, such as the 9-point hedonic scale. In this scale, panelists select an expression that aligns with their perception and acceptance of the product. The utilization of such scales enables the conversion of responses into numerical values, such as 1 for "dislike extremely" to 9 for "like extremely." This evaluative approach swiftly furnishes information about the potential success and appeal of a newly developed product. Furthermore, hedonic tests can unveil details about various consumer clusters based on factors such as product type, textures, composition, and more. While these results offer valuable insights into the rationale behind liking or disliking a product, the hedonic technique comes with certain limitations. Firstly, a sufficient number of panelists, ideally representative of the target consumers is needed. The testing environment and circumstances should ideally mirror real-life situations in which consumers would encounter the product. Typically, employing more than 60 representative consumers is standard practice. It is important to note that the outcomes of this test do not necessarily indicate consumer purchase intention, as various factors beyond liking come into play. Assessing purchase intention often demands a more extensive participant pool, typically exceeding 100 individuals, to capture a more comprehensive and accurate understanding of consumer behavior. Currently, the combination of affective and descriptive sensory technologies is often used. This strategic integration allows for leveraging the advantages of each technique while mitigating their respective disadvantages. It aids in gaining insights into consumer preferences and acceptance (affective) and discerning what attributes should be enhanced, maintained, or adjusted during the formulation or preparation of products. However, traditional sensory analyses have displayed limitations, often neglecting certain aspects within the intricate realm of consumer-product interactions. These interactions extend beyond the conscious responses captured on a liking scale, as external stimuli play a role in influencing decisions and the level of acceptance of a food product. To truly comprehend consumers' preferences for a product, it becomes essential to delve into their broader needs and constraints, encompassing factors like purchasing power, prices of both fresh and processed products, product quality, health connotations (such as fat content and additives), and the context of consumption. Recognizing these aspects is crucial for overcoming the limitations inherent in traditional sensory techniques. To address these challenges, innovative sensory and consumer research techniques have been developed, aiming to provide a more comprehensive understanding of the complex dynamics involved in consumer-product interactions (Ruiz-Capillas et al., 2021). Recruitment of consumer panelists is essential to conduct hedonic assessments for meat and meat products. Depending on the project's objectives, various demographic and socio-economic criteria, such as age, gender, household size, income, area of residence, usage patterns (frequent vs. light users), and attitudes, are taken into consideration. The goal is typically to engage consumers who regularly use the products being tested. Given that hedonic ratings from consumers tend to exhibit greater variability than those from trained panelists, large sample sizes are necessary to establish the significance of product effects (Mörlein, 2019).

### Qualitative Affective Tests

Qualitative tests provide a subjective response, usually from consumers. Such methods allow consumers to talk about their feelings regarding the sensory properties of a set of products. Some examples of qualitative tests are: focus groups or panels; mini groups; dyads and triads; and one-on-one interviews (AMSA, 2016). For example, Kennedy et al. (2004a) applied the focus group procedure to investigate sensory drivers of liking by consumers. The authors carried out the study with six focus groups (4–8 participants, mixed in terms of sex) and each focus group was facilitated by a moderator and took around 30–45 min. On the other hand, Piochi et al. (2023) used the focus group method before the sensory test to select the attributes to be included in the subsequent sensory evaluation made by consumers; the authors recruited 8 subjects (balanced in terms of sex and with an average age of 38 years old).

### Quantitative Affective Tests

Quantitative hedonic tests play a crucial role in assessing consumers' sensory perception of products, employing a set of questions to measure preferences, likings, and impressions across various sensory attributes. These tests can take different forms, such as central location testing (**CLT**) with pre-recruited participants, non-pre-recruited tests like mall intercept tests, or home use testing (**HUT**). When the goal of consumer evaluation is to identify the preferred product, preference testing is conducted. If the objective is to gauge how well a product is liked, acceptance tests are employed. Hedonic scales, typically ranging from like to dislike, are utilized to quantify the degree of acceptability. To delve deeper into specific attributes, additional scales like Just-About-Right (**JAR**) and intensity scales are employed. JAR scales help determine when an attribute is perceived as too high or too low, while intensity scales provide insights into the strength or weakness of a particular attribute. This multifaceted approach allows for a comprehensive understanding of consumers' perceptions and preferences in the realm of sensory testing (AMSA, 2016). Kennedy et al. (2004b) assessed the liking of poultry meat in different conditions: lab-controlled environment (37 consumers) and at home (30 consumers). Saha et al. (2009b) carried out a sensory evaluation on breast fillets with 63 consumers that had to evaluate liking on a 9-point hedonic scale and the adequacy of perceived intensity of tenderness, moistness, overall flavor, and saltiness of a 5-point JAR scale. Napolitano et al. (2013) carried out a consumer sensory test involving 150 subjects (recruiting procedure based on age and consumption frequency of chicken and organic products), who had to rate their liking after tasting the meat under blind and informed conditions. Toomer et al. (2019) assessed liking on a 9-point hedonic scale and the adequacy of color and flavor attributes of chicken breasts by 100 consumers; while Damaziak et al. (2019) investigated liking of chicken breast and legs comparing two groups of consumers: normal sighted (132 subjects) and blind people (103 subjects). Xu et al. (2020) applied a combination of descriptive tests (CATA and RATA) and hedonic questions to assess the sensory profile of chicken meat as well as their liking and emotional responses by consumers (61 subjects). A similar procedure has been applied also by Escobedo del Bosque et al. (2022) with 95 consumers divided into three groups.

## CONCLUSIONS

From the literature review and critical discussion, the relevant aspects primarily concern sample preparation; indeed, in the case of poultry meat, a pre-treatment such as cooking is generally required for sensory evaluation, aligned with the purpose of the test. As discussed, methods like grilling or baking, which can generate aromatic compounds due to the Maillard reaction, may not be suitable if the goal is to evaluate subtle sensory differences that strong cooking can mask; in such cases, sous-vide cooking might be much more appropriate. Additionally, the serving temperature of the sample is also crucial for reliable results and comparisons and when, as often happens, this is not specified in the literature, this is certainly a gap. Serving a sample at an inappropriate temperature could introduce biases in the results; for instance, tasters might struggle to identify flavor nuances if the sample is too cold, or it might be challenging to taste if it is too hot. Furthermore, depending on the study's objective, it is necessary to select the method that best suits the requirements. For instance, if the goal is to assess the existence of a difference between two or more samples, such as related to dietary supplements in chicken, applying a discriminative test is necessary. On the other hand, if further investigation into this sensory difference is desired, applying a descriptive method, whether rapid or not, could be useful. It would not be practical to use a time-consuming method like QDA for a single evaluation; in this case, a rapid method could be applied. Conversely, if the intention is to establish an enduring sensory quality control system, training a panel is essential, making it advisable to apply a method like QDA. Finally, if the aim is to investigate liking, it is necessary to apply an effective method, which can be combined with a descriptive method like CATA if there is a desire to explore consumers' sensory perception in terms of perceived sensory attributes. However, regardless of the type of sensory test applied, given the importance of ethical considerations, it is essential to always take into account the importance of obtaining the necessary ethical approvals to conduct a sensory evaluation before starting the test. In conclusion, this review is presented as a first guideline for the sensory evaluation of chicken meat. A further step could be the compiling of validated and widely recognized procedures, in particular for cooking and serving samples, reporting also potential relations between the sensory properties and specific attributes measured by instrumental methods, such as texture, flavor or color.

## DISCLOSURES

The authors declare no conflicts of interest.

Declaration of AI and AI-Assisted Technologies in Writing: During the preparation of this work the author(s) used Chat GPT in order to improve readability and language (e.g., reformulation of sentences). After using this tool/service, the author(s) reviewed and edited the content as needed and take(s) full responsibility for the content of the publication.
